# The Small Molecule BIBR1532 Exerts Potential Anti-cancer Activities in Preclinical Models of Feline Oral Squamous Cell Carcinoma Through Inhibition of Telomerase Activity and Down-Regulation of TERT

**DOI:** 10.3389/fvets.2020.620776

**Published:** 2021-01-20

**Authors:** Gennaro Altamura, Barbara degli Uberti, Giorgio Galiero, Giovanna De Luca, Karen Power, Luca Licenziato, Paola Maiolino, Giuseppe Borzacchiello

**Affiliations:** ^1^Department of Veterinary Medicine and Animal Productions, University of Naples Federico II, Naples, Italy; ^2^Istituto Zooprofilattico Sperimentale del Mezzogiorno, Portici, Naples, Italy

**Keywords:** BIBR1532, telomerase activity, TERT, EGFR, MMP, cancer, feline oral squamous cell carcinoma

## Abstract

Expression of telomerase reverse transcriptase (TERT) and telomerase activity (TA) is a main feature of cancer, contributing to cell immortalization by causing telomeres dysfunction. BIBR1532 is a potent telomerase inhibitor that showed potential anti-tumor activities in several types of cancer, by triggering replicative senescence and apoptosis. In a previous work, we detected, for the first time, TERT expression and TA in preclinical models of feline oral squamous cell carcinoma (FOSCC); therefore, we aimed at extending our investigation by testing the effects of treatment with BIBR1532, in order to explore the role of telomerase in this tumor and foreshadow the possibility of it being considered as a future therapeutic target. In the present study, treatment of FOSCC cell lines SCCF1, SCCF2, and SCCF3 with BIBR1532 resulted in successful inhibition of TA, with subsequent cell growth stoppage and decrease in cell viability. Molecular data showed that up-regulation of cell cycle inhibitor p21, unbalancing of Bax/Bcl-2 ratio, and down-regulation of survival gene Survivin were mostly involved in the observed cellular events. Moreover, BIBR1532 diminished the expression of TERT and its transcriptional activator cMyc, resulting in the down-regulation of epidermal growth factor receptor (EGFR), phospho-ERK/ERK ratio, and matrix metalloproteinases (MMPs)-1/-2 and−9, likely as a consequence of an impairment of TERT extra-telomeric functions. Taken together, our data suggest that BIBR1532 exerts multiple anti-cancer activities in FOSCC by inhibiting telomerase pathway and interfering with signaling routes involved in cell proliferation, cell survival, and invasion, paving the way for future translational studies aimed at evaluating its possible employment in the treatment of this severe tumor of cats.

## Introduction

Telomerase is a holoenzyme that extends telomeres, the terminal DNA sequences of chromosomes (1). This function is carried out mostly by its catalytic subunit, the telomerase reverse transcriptase (TERT), which works by adding TTAGGG repeats to telomeric DNA by using an internal RNA template (TERC) (1).

In somatic cells, TERT gene is not transcribed, and telomerase is not active; therefore, telomeric ends shorten at each cell duplication until, after a scheduled number of replications, they reach a critical point of erosion (1). The cell machinery senses this event like a severe DNA damage and thus activates p53-dependent molecular pathways that result in replicative senescence and cell death, including p21-induced cell cycle arrest and apoptosis through unbalancing of genes belonging to the Bcl-2 family, such as the pro-apoptotic gene Bax and the anti-apoptotic gene Bcl-2 (1).

Instead, cancer cells are prone to maintain a highly proliferative rate; therefore, it is not surprising that TERT expression and telomerase activity (TA) represent a common feature of most of human malignancies (2). A main player in the activation of this pathway in tumor cells is the cMyc oncogene that, among its multiple transforming properties, works as a positive transcriptional regulator of TERT gene, constituting a key signaling axis in cancer (3, 4); once this is switched on, active telomerase triggers cell immortalization by preventing telomeres shortening, thus resulting in the abrogation of replicative senescence (1). However, there is increasing evidence that, beyond its canonical activity, TERT displays some extra-telomeric functions, such as transcriptional regulation of genes with a relevant role in oncogenic pathways (2). For instance, TERT is a positive regulator of epidermal growth factor receptor (EGFR), a membrane tyrosine kinase that activates signal transduction cascades that results in cell proliferation and inhibition of apoptosis (2, 5, 6). And yet, it modulates the expression of genes influencing cell migration and invasiveness, such as matrix metalloproteinases (MMPs)-1/-2/-9, thus playing a role in epithelial–mesenchymal transition and metastasis (2, 7–9). For these reasons, TERT is actually considered a central regulator of the hallmarks of cancer (2).

Due to this multilevel role in carcinogenesis, different strategies are under investigations for telomerase inhibition, such as gene silencing through siRNA or treatment with small molecules (10). BIBR1532 is a non-nucleosidic, non-peptidic inhibitor of telomerase that blocks enzymatic activity by binding to the active site of TERT (11). Treatment of many cancer cell types with this drug has been shown to efficiently repress TA, leading to growth arrest and cell death (10). Moreover, independently from its activity as functional telomerase inhibitor, BIBR1532 induces the down-regulation of TERT expression, which in turn further weakens TA and provides additional anti-cancer effects linked to impairment of its extra-telomeric functions (8, 12). In addition, BIBR1532 displayed potential efficacy as adjuvant in combination with other anti-cancer agents and radiotherapy in lung and breast cancer as well as acute promyelocytic leukemia (13–15). Therefore, BIBR1532 may be potentially an ideal compound for cancer therapy.

Telomerase inhibition represents a promising therapeutic approach for human oral squamous cell carcinoma (hOSCC), where the role of this enzyme is prominent (16–18). Then, there is increasing interest in veterinary oncology for studying telomerase in animal counterparts, particularly in canine and feline OSCC (FOSCC) (19–21). FOSCC is a malignant tumor characterized by highly aggressive behavior, frequent local invasion, metastasis, high rate of recurrence, and often poor prognosis (22). Indeed, surgery, radiotherapy, and chemotherapy are possible treatment strategies; however, no 100% efficient therapy is currently available (23). A recent study of our group demonstrated the expression of cMyc, TERT, and TA, along with the expression of MMP-1/-2/-9 in two validated preclinical models of FOSCC, namely, SCCF2 and SCCF3 (24–26). With the present work, we aimed at expanding our investigation by testing the effects of the specific inhibitor BIBR1532 in SCCF2, SCCF3, and, importantly, in an additional validated tumor-derived cell line named SCCF1, in order to investigate the role of telomerase pathway in these tumors and explore the potential anti-cancer activities of this drug in FOSCC (27).

## Materials and Methods

### Cell Lines, Cell Culture, and Treatments

FOSCC cell lines SCCF1, SCCF2, and SCCF3 developed in the Rosol laboratory are a kind gift from Professor T.J. Rosol (The Ohio State University) and were cultured as previously described (24, 25, 27). A stock solution of BIBR1532 (Selleckchem #S1186) at 10 mM was prepared by dissolving the compound in dimethylsulfoxide (DMSO, Bioshop #67-68-5) and stored at −80°C in aliquots to be thawed immediately before use. For treatment, cells were plated at 1 × 10^5^ density in 6-well plates, and after 24 h, BIBR1532 diluted at 25, 50, and 100 μM in culture medium was added to wells. In control plates, drug was replaced with the same amount of DMSO for each dose. After 48 h, wells were read under ZOE Fluorescent Cell Imager (Bio-Rad Laboratories) for scanning and phase contrast photography, and then cells were harvested by trypsinization and analyzed as described below.

### Trypan Blue Exclusion Assay for Cell Count and Viability

Cell pellets from treatments at 25, 50, and 100 μM for 48 h were resuspended in phosphate buffered saline (PBS, Corning #21-040-CV), and then equal amounts of cell suspension and 0.4% trypan blue (Lonza #17-942E) were mixed, incubated for 1–2 min at room temperature (rt), and counted by the use of Cell Counter (Bio-Rad). Changes in cell viability and growth at each dose of treatment with BIBR1532 were calculated and expressed as percentage compared with the respective control plate treated with DMSO set as 100%.

### Telomeric Repeat Amplification Protocol Assay

TA in FOSCC cell lines treated at 25, 50, and 100 μM for 48 h was assessed by a telomeric repeat amplification protocol (TRAP) assay by using TRAPeze® Telomerase Detection Kit (Merck #S7700) as previously described (26). Reaction products were run on non-denaturing 15% polyacrylamide gel electrophoresis (PAGE). Gels were stained with GelStar™ Nucleic Acid Gel Stain (Lonza #50535) and read through the ChemiDoc gel scanner (Bio-Rad). For quantification, densitometric analysis was performed by using Image Lab software (Bio-Rad), and TA was calculated as the ratio between the telomerase ladders and the 36-base pair internal control. Decrease in TA at each dose was expressed as percentage compared with the respective control plate treated with DMSO set as 100%.

### Western Blotting

Total protein extraction, protein quantification, sodium dodecyl sulfate (SDS)-PAGE, and Western blotting (WB) on cells treated at 50 or 100 μM for 48 h were performed as reported elsewhere (28). Detection of TERT, MMP-1, MMP-2, MMP-9, β-actin, and tubulin protein bands in SCCF cells was obtained as described previously (26). Detection of ERK, phospho-ERK, and Caspase 3 was performed by using antibodies and protocols described in previous work, anti-EGFR (Thermo Fisher Scientific #MS-378-P0) was also applied overnight at 4°C at 1:1,000 dilution, and HeLa whole cell lysate was loaded along with feline samples to ensure the reactivity of these three antibodies and the identity of the bands in SCCF cells (29, 30). Goat anti-mouse (GE Healthcare #LNA931V/AH) and donkey anti-rabbit (Bethyl Laboratories #A-120-108P) secondary antibodies conjugated with horseradish peroxidase were applied for 1 h at rt, and protein bands were visualized by enhanced chemiluminescence (ECL, Bio-Rad) at ChemiDoc gel scanner (Bio-Rad). Densitometric analysis for protein quantization was achieved by using Image Lab software (Bio-Rad). Protein expression levels were normalized to β-actin, and phosphorylation status of ERK was calculated as densitometric ratio pERK/ERK. Changes in protein expression upon treatments at each dose were calculated and expressed by comparison with the respective control plate treated with DMSO set as 1.

### RNA Extraction, Reverse Transcription, and Real-Time Quantitative PCR

Total RNA extraction, reverse transcription (RT) to cDNA, and real-time quantitative PCR (qPCR) for feline EGFR, TERT, and cMyc were performed on cells treated at 50 μM for 48 h, as previously described (26, 31). For amplification of feline Survivin, p21, Bax, and Bcl-2 genes in cells treated at 25, 50, and 100 μM for 48 h, primers sets reported elsewhere were employed (32–34). Gene expression results were normalized for feline β2-microglobulin (β2MG), and data were generated according to the 2^−ΔΔCt^ method by using Bio-Rad CFX Manager software (35). Changes in gene expression were calculated as relative mRNA levels at each dose with respect to DMSO control plate set as 1.

### Statistical Analysis

For statistical analysis, Student's *t*-test was performed using SPSS 17.0 software (SPSS Inc.), and differences were considered to be statistically significant for ^*^P < 0.05 or ^**^P < 0.01.

## Results

### BIBR1532 Inhibits TA in FOSCC Cell Lines

**Figure 1:**
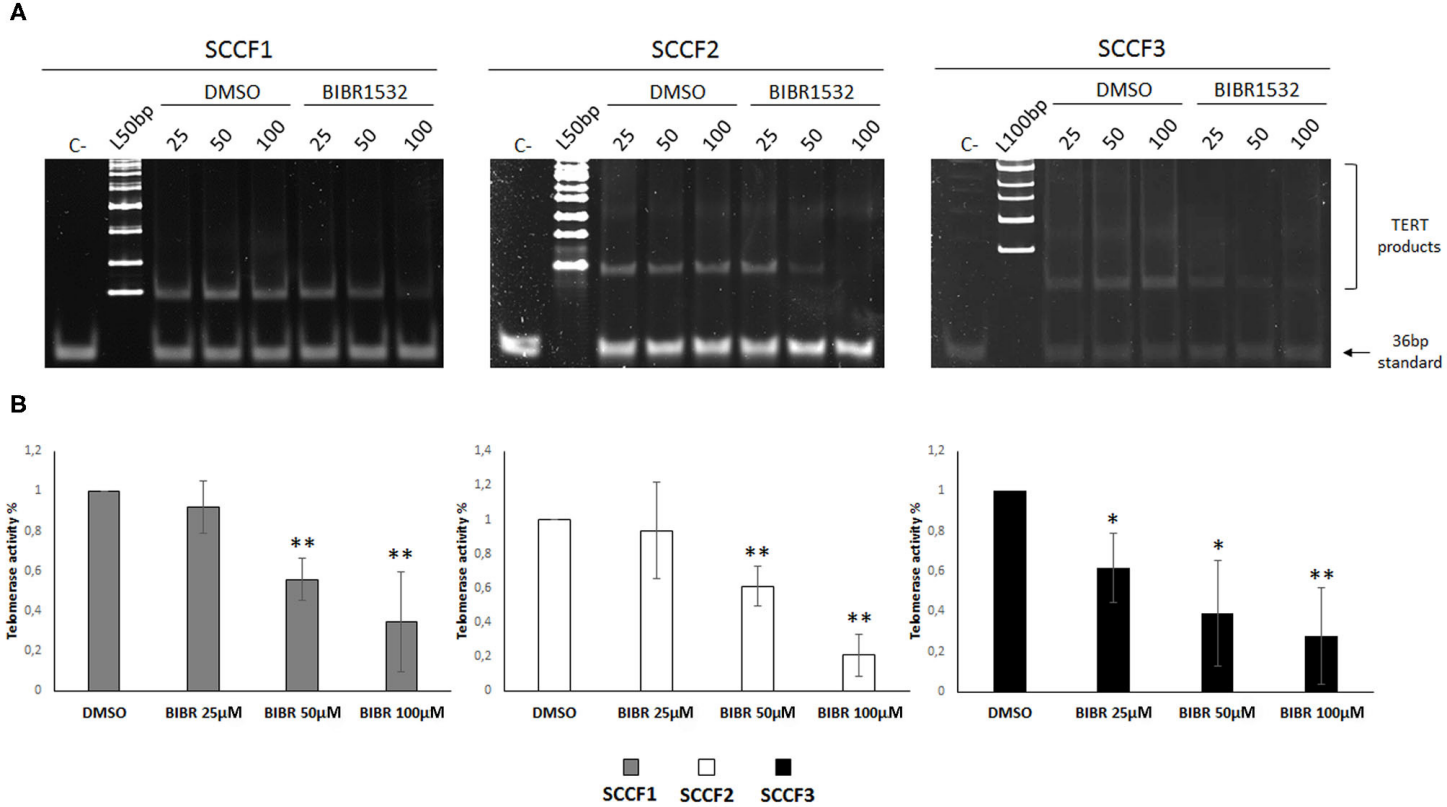
BIBR1532 down-regulates telomerase activity (TA) in a dose-dependent manner in FOSCC cell lines. **(A)** SCCF1, SCCF2, and SCCF3 were treated with BIBR1532 at 25, 50, and 100 μM *vs* DMSO and analyzed by telomeric repeat amplification protocol (TRAP) assay. Representative gels out of at least three independent experiments are illustrated (C–: negative control, sample with no lysate; L100bp: 100 bp DNA ladder, the first band from the bottom is 100 bp; L50bp: 50 bp DNA ladder, the first band from the bottom is 50 bp). **(B)** Quantification of TA by densitometric analysis of gel scans. Data were calculated as the ratio between TERT products ladder and 36 bp internal standard and represent the mean ± standard deviations of at least three independent experiments. Changes in TA were calculated and expressed as % for each BIBR1532 dose compared with its respective DMSO control set as 100% (statistically significant, *P < 0.05; **P < 0.01).

BIBR1532 blocks TA by specifically limiting the amount of nucleotide repeats available for substrate elongation; this activity has been detected in numerous preclinical models of cancer; however, it has never been tested in FOSCC cell lines (10). In order to define the effects of BIBR1532 on TA in FOSCC, SCCF1, SCCF2, and SCCF3 were treated at 25, 50, and 100 μM for 48 h and subjected to TRAP assay. The results showed readily detectable TA at control conditions in SCCF1, SCCF2, and SCCF3 (Figure 1A), and importantly, gel scans and quantitative analysis demonstrated reduction of TA with a dose-dependent trend in BIBR1532-treated cells in all of the three cell lines (Figures 1A,B). However, SCCF3 appeared to be sensitive already at the lower dose (25 μM), whereas SCCF1 and SCCF2 showed significant telomerase inhibition from 50 μM onward (Figures 1A,B).

### BIBR1532 Affects Cell Growth, Viability, and Morphology in SCCF1, SCCF2, and SCCF3

**Figure 2:**
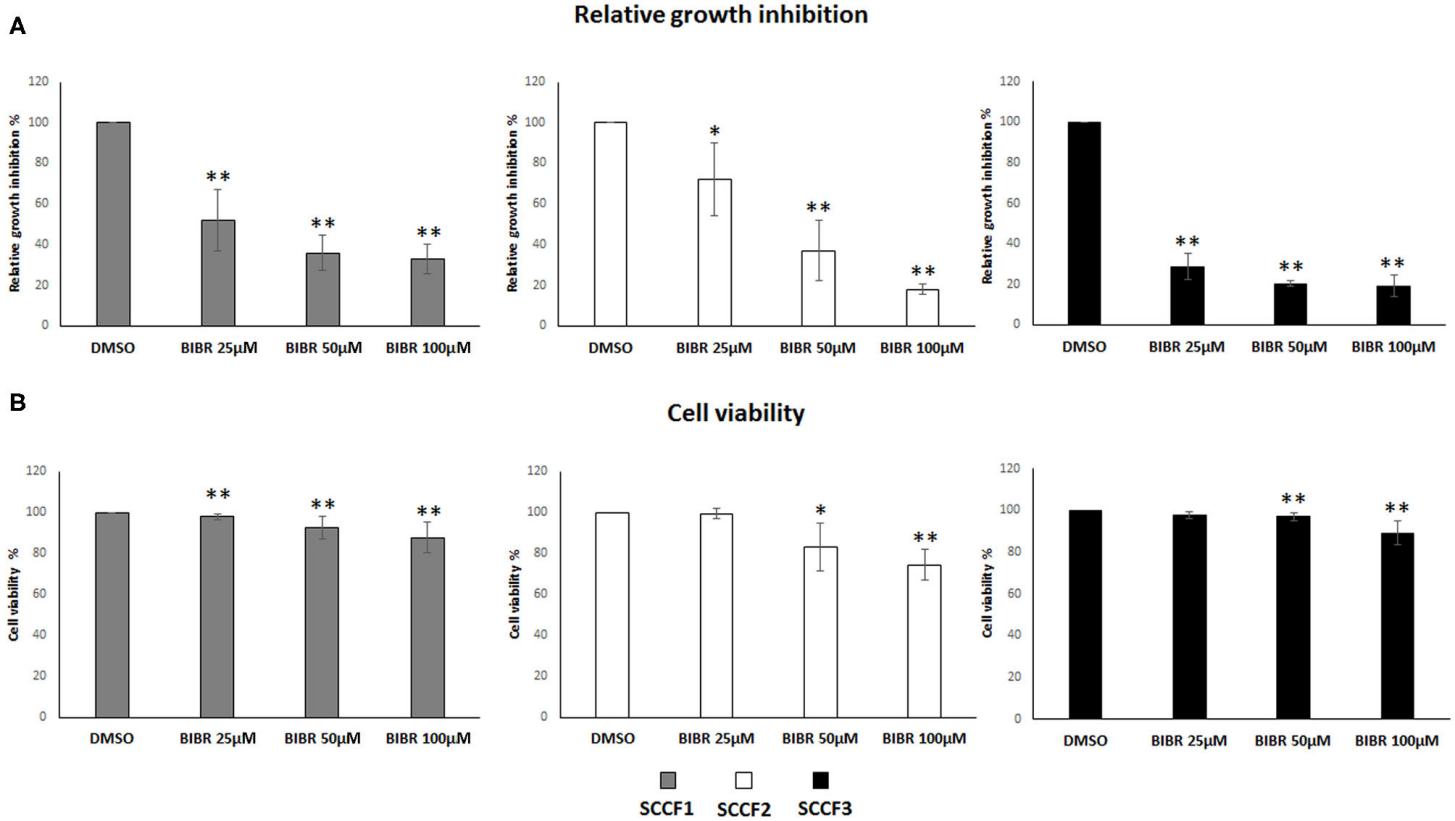
BIBR1532 inhibits cell growth and decreases cell viability in FOSCC cell lines. **(A)** SCCF1, SCCF2, and SCCF3 were treated with BIBR1532 at 25, 50, and 100 μM *vs* DMSO and analyzed by cell counting. The inhibitory effect on cell growth of each dose of BIBR1532 was calculated and expressed as % of decrease compared with its respective DMSO control set as 100%. Mean ± standard deviations (SD) from at least four independent experiments are shown. **(B)** Cell viability assessed by trypan blue exclusion assay. The effect of each dose of was calculated and expressed as % of decrease compared with its respective DMSO control set as 100%; the plots represent mean ± SD from at least four independent experiments (statistically significant, *P < 0.05; **P < 0.01).

Then, to investigate the impact of telomerase inhibition by BIBR1532 on these cell lines, cell growth and viability were evaluated under the same experimental conditions. The treatment induced growth inhibition in SCCF1, SCCF2, and SCCF3 in a dose-dependent manner (Figure 2A); notably, this effect was more marked in SCCF3 than in the other cell lines, and this was already noticeable at the lowest dose (Figure 2A). Moreover, BIBR1532 caused a dose-dependent decrease in cell viability in SCCF2 and, although slightly, in SCCF1, whereas in SCCF3, a mild reduction was yielded only at 100 μM (Figure 2B).

To further evaluate the possible effects of BIBR1532 on cell morphology, cells were scanned by phase contrast microscope. In agreement with growth arrest data, the number of cells observable in treated wells decreased in a dose-dependent manner, and interestingly, morphologic changes were also visible upon BIBR1532 incubation in all cell lines: unlike untreated cells, growth-arrested SCCFs became flattened, enlarged, with intercellular bridges and increased granularity, often multi-nucleated and had vacuolated cytoplasm (Supplementary Figures 1A–C).

### Molecular Changes Associated With Growth Arrest and Cell Death Induced by BIBR1532

**Figure 3:**
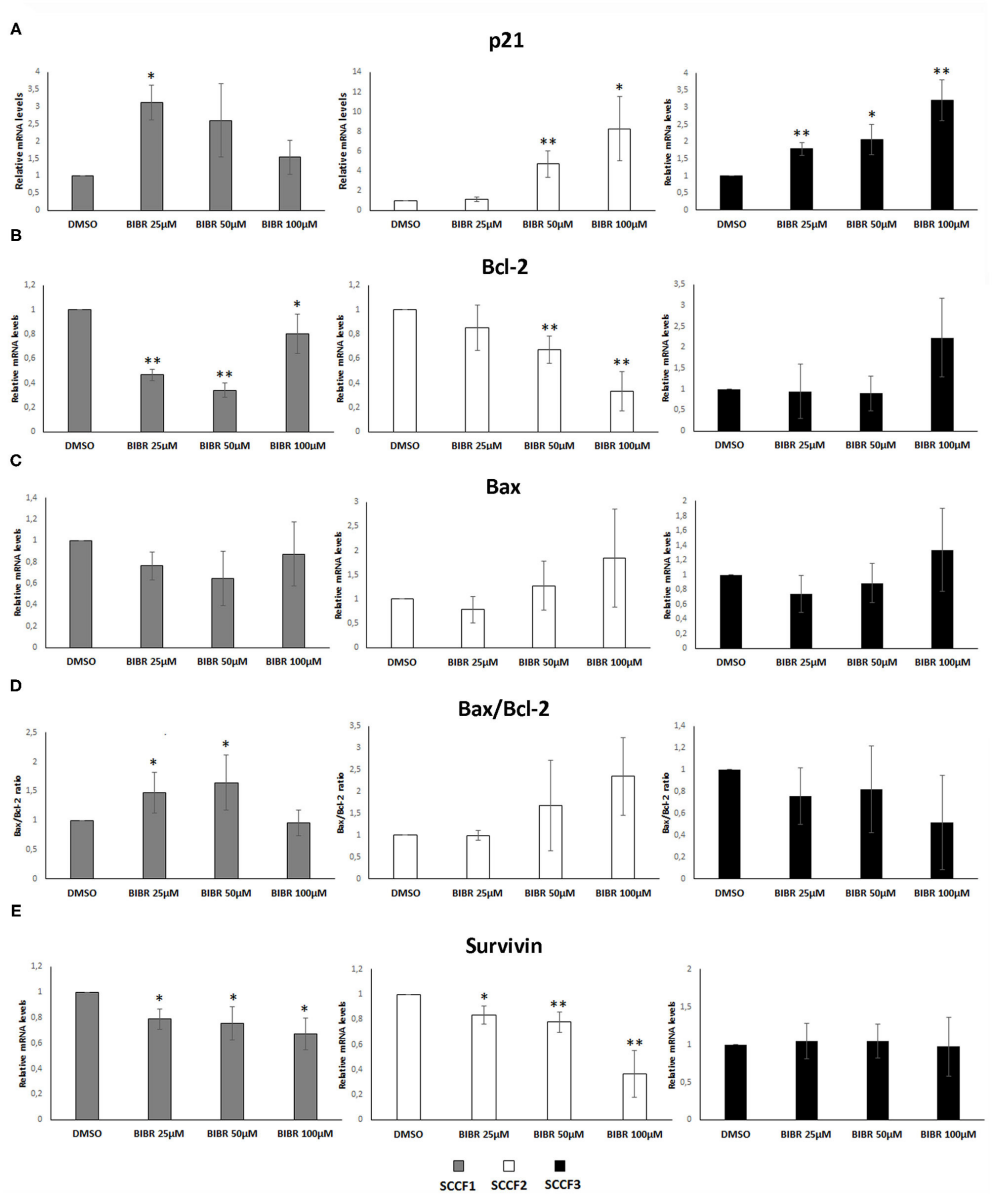
Gene expression changes in p21, Bcl-2, Bax, and Survivin in FOSCC cell lines treated with BIBR1532. SCCF1, SCCF2, and SCCF3 were treated with BIBR1532 at 25, 50, and 100 μM *vs* DMSO and analyzed by qPCR for p21 **(A)**, Bcl-2 **(B)**, and Bax **(C)**. Bax/Bcl-2 transcriptional ratio **(D)** and Survivin gene expression **(E)** were also calculated as a measure of activated apoptosis. Data were normalized for β2-microglobulin and expressed as relative quantization by the 2^−ΔΔ*Ct*^ method. Changes in relative mRNA levels for each dose of BIBR1532 were calculated and compared with their respective DMSO control set as 1. Plots represent the mean ± standard deviations of at least three repeated, independent experiments performed in technical triplicate (statistically significant, *P < 0.05; **P < 0.01).

Treatment with BIBR1532 of different cancer cell lines causes cell cycle arrest and apoptosis through activation of transcriptional pathways triggered by telomeres dysfunction, such as the increase of cell cycle inhibitor p21 and the unbalancing of Bax/Bcl-2 ratio (36–38). To further characterize whether these molecular changes caused by BIBR1532 would also contribute to cellular phenomena observed above in SCCF cells, qPCR experiments for p21, Bax, and Bcl-2 were conducted upon treatment at 25, 50, and 100 μM for 48 h (Figure 3).

In SCCF1, the highest increase of p21 gene expression was yielded at 25 μM, and this was followed by a gradual decrease at 50 and 100 μM, whereas a dose-dependent effect was observed in treated SCCF2 and SCCF3 cells (Figure 3A); however, SCCF3 but not SCCF2 showed increase of p21 already at the lowest dose (25 μM), although the fold of up-regulation at increasing doses (50 and 100 μM) was higher in SCCF2 with respect to SCCF3 (Figure 3A).

When analyzing Bax and Bcl-2 expression and their molecular ratio, the following results were observed: SCCF1 showed a dose-related reduction of Bcl-2 at 25 and 50 μM but not at 100 μM (Figure 3B); however, it was not accompanied by Bax augmentation; therefore, Bax/Bcl-2 ratio was mildly and gradually increased only at 25 and 50 μM to then decrease to control conditions at 100 μM (Figures 3C,D). In SCCF2 cell line, a dose-dependent decrease of Bcl-2 along with an increase in Bax mRNA levels was yielded, leading to an unbalancing of their molecular ratio in favor of Bax and thus apoptosis, which was particularly impressive at 100 μM (Figures 3B–D). SCCF3 showed no significant trends in Bax neither in Bcl-2 levels; therefore, no change of possible biological relevance could be observed in Bax/Bcl-2 ratio (Figures 3B–D).

BIBR1532 affects cell survival in leukemia cell lines by reducing the expression of the Survivin gene; therefore, its mRNA levels were also investigated in treated SCCF cells (37, 38). In SCCF1 and SCCF2, Survivin expression decreased in a dose-dependent manner, whereas no change could be observed in treated SCCF3 at any drug concentration (Figure 3E). Despite the variable molecular responses, WB analysis for cleavage of Caspase 3 confirmed the occurrence of apoptosis at 100 μM in all of the three cell lines, in agreement with cell viability data (Supplementary Figure 2).

### BIBR1532 Down-Regulates cMyc and TERT Expression

**Figure 4:**
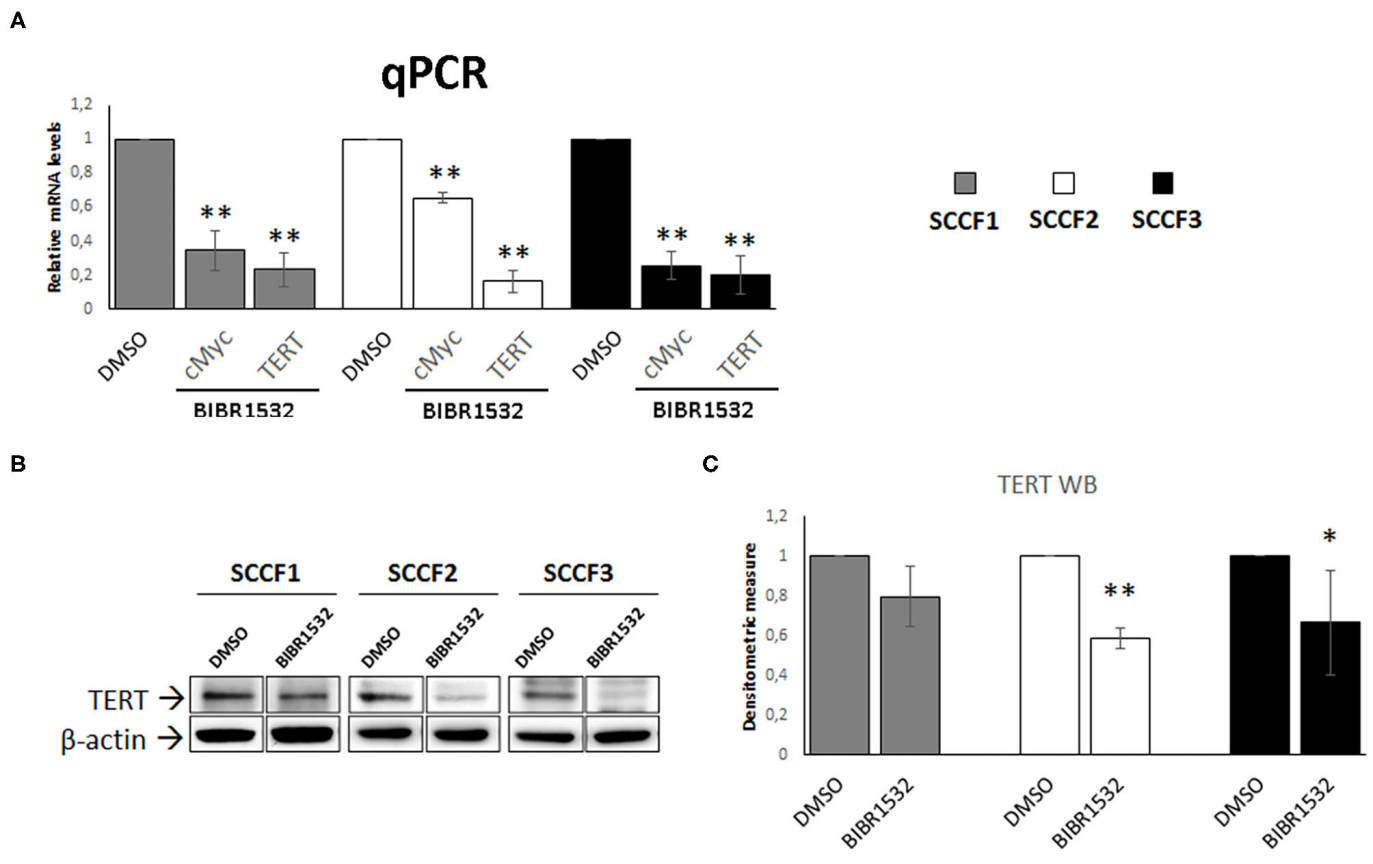
BIBR1532 down-regulates cMyc and TERT in FOSCC cell lines. **(A)** SCCF1, SCCF2, and SCCF3 were treated with BIBR1532 at 50 μM *vs* DMSO and analyzed by qPCR for TERT and cMyc gene expression. Data were normalized for β2-microglobulin as housekeeping gene and expressed as relative quantization by the 2^−ΔΔ*Ct*^ method. Changes in relative mRNA levels were calculated for SCCF1, SCCF2, and SCCF3 treated with BIBR1532 compared with their respective DMSO control set as 1. Plots represent the mean ± standard deviations (SD) of at least three repeated, independent experiments performed in technical triplicate. **(B)** A representative Western blotting (WB) gel for TERT protein expression in BIBR1532- vs. DMSO-treated cells. The blot was stripped and reprobed for β-actin to ensure comparable protein loading and allow normalization. For each cell line, boxes are cut from the same gel at the same exposure time and properly aligned according to molecular standards loaded onto the gel. Full scans from original gels are shown in Supplementary Figure 3. **(C)** Mean densitometric values normalized for β-actin expression ± SD from at least three repeated, independent WB experiments. Changes in protein levels were calculated for SCCF1, SCCF2, and SCCF3 treated with BIBR1532 compared with their respective DMSO control set as 1 (statistically significant, *P < 0.05 and **P < 0.01).

In addition to the canonical inhibitory effects on TA, treatment with BIBR1532 has been shown to down-regulate the expression of cMyc in a series of cancer cell lines, resulting in decreased TERT transcription (36–39). Consistently, when analyzing cMyc and TERT expression, we found a decrease in their relative mRNA levels in BIBR1532- (50 μM for 48 h) vs. DMSO-treated cells in SCCF1, SCCF2, and SCCF3 (Figure 4A). Down-regulation of TERT was further confirmed at protein level by WB with a specific antibody, followed by densitometric analysis (Figures 4B,C).

### Effects of BIBR1532 on EGFR Pathway

**Figure 5:**
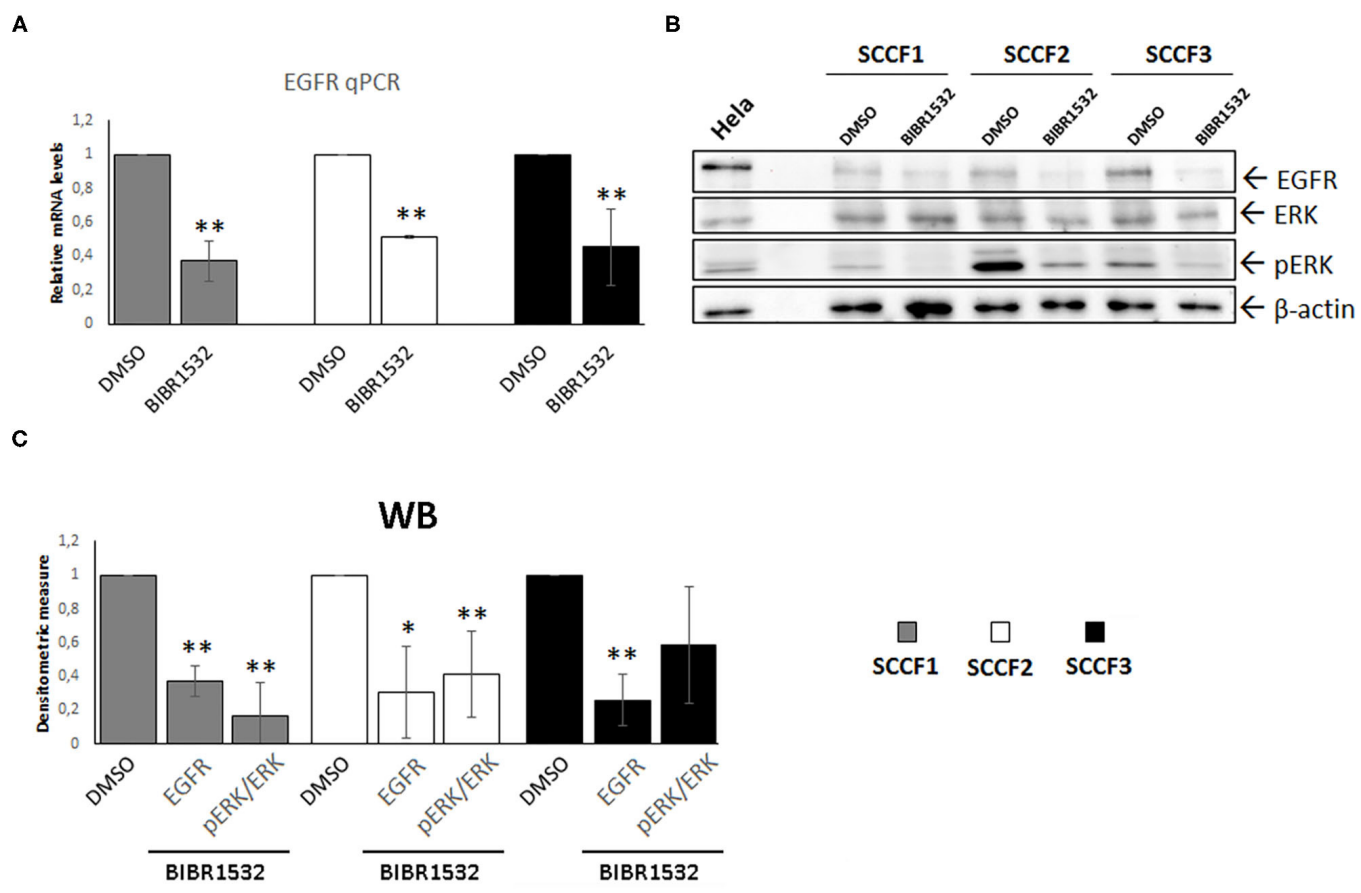
BIBR1532 down-regulates EGFR pathway in FOSCC cell lines. **(A)** SCCF1, SCCF2, and SCCF3 were treated with BIBR1532 50 μM vs. DMSO and analyzed by qPCR for EGFR gene expression. Data were normalized for β2-microglobulin and expressed as relative quantization by the 2^−ΔΔ*Ct*^ method. Changes in relative mRNA levels were calculated for SCCF1, SCCF2, and SCCF3 treated with BIBR1532 compared with their respective DMSO control set as 1. Plots represent the mean ± standard deviations (SD) of at least three repeated, independent experiments performed in technical triplicate. **(B)** Representative WB for EGFR, ERK, and pERK in BIBR1532- vs. DMSO-treated cells. HeLa whole cell lysate run along with feline samples confirmed the identity of the bands. The blot was stripped and reprobed for β-actin to ensure comparable protein loading and allow normalization. **(C)** Densitometric values of EGFR normalized for β-actin and densitometric ratio pERK/ERK; data are expressed as mean ± SD from at least three repeated, independent experiments. Changes in protein levels were calculated for SCCF1, SCCF2, and SCCF3 treated with BIBR1532 compared with their respective DMSO control set as 1 (statistically significant, *P < 0.05 and **P < 0.01).

Beyond its role as catalytic subunit of telomerase, TERT exerts several extra-telomeric functions; among these, transcriptional regulation of EGFR is of particular relevance, especially in cancer (2, 5, 6). Here, EGFR expression was readily detected at control conditions in SCCF1, in agreement with a previous study and, for the first time, in SCCF2 and SCCF3 cells (Figures 5A,B) (40). Notably, treatment with BIBR1532 at 50 μM for 48 h caused the down-regulation of EGFR mRNA and protein levels in all of the three cell lines as detected by qPCR and WB, respectively, likely due to the decreased expression of TERT (Figures 5A–C). Signal transduction pathways downstream of EGFR result in phosphorylation/activation of mitogen-activated protein kinase ERK (41). Consistently, in treated SCCFs, reduction of EGFR was associated with a marked decrease of activated ERK (pERK), as detected by WB and confirmed by measuring pERK/ERK densitometric ratio (Figures 5B,C).

### BIBR1532 Down-Regulates MMP-1, MMP-2, and MMP-9

**Figure 6:**
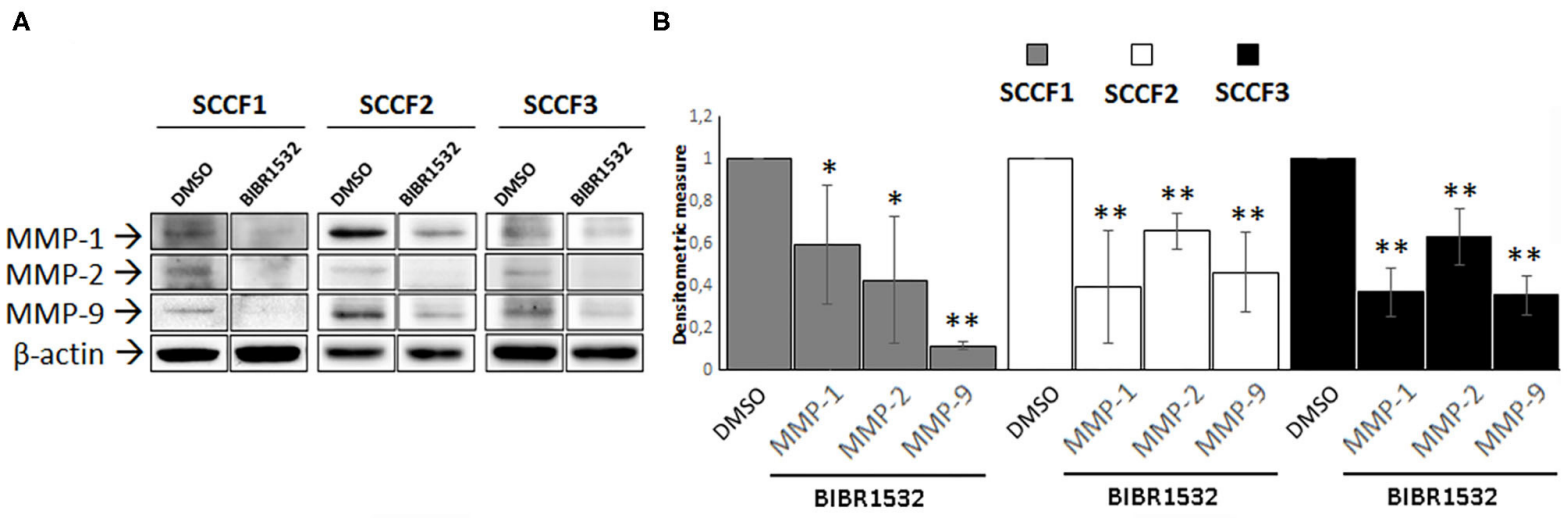
BIBR1532 down-regulates MMP-1/-2/-9 in FOSCC cell lines. **(A)** SCCF1, SCCF2, and SCCF3 were treated with BIBR1532 50 μM vs. DMSO and analyzed by Western blotting (WB) for MMP-1/-2/-9. Representative WB experiments showing lower MMP-1/-2/-9 expression at protein level in BIBR1532- vs. DMSO-treated cells are shown. Blots were stripped and reprobed for β-actin to ensure comparable protein loading and allow normalization. For each cell line, boxes are cut from the same gel at the same exposure time and properly aligned according to molecular standards loaded onto the gel. Full scans from original gels are shown in Supplementary Figure 4. **(B)** Mean densitometric values normalized for β-actin expression ± SD from at least two repeated, independent experiments. Changes in MMPs protein levels were calculated for SCCF1, SCCF2, and SCCF3 treated with BIBR1532 compared with their respective DMSO control set as 1 (statistically significant, *P < 0.05 and **P < 0.01).

Treatment with BIBR1532 decreases the protein expression of several MMPs in different types of cancer cells, through down-regulation of TERT (8, 9). To address whether this might occur also in SCCF1, SCCF2, and SCCF3, WB experiments for MMP-1, MMP-2, and MMP-9 and densitometric analysis were performed upon treatment at 50 μM for 48 h (Figures 6A,B). The results demonstrated that incubation with the drug yielded a decrease in MMP-1, MMP-2, and MMP-9 protein levels compared with control conditions, in all of the three cell lines (Figure 6).

## Discussion

With the present study, we aimed at expanding our ongoing investigation on the role of TERT and TA in FOSCC by treating tumor cell lines with the specific inhibitor BIBR1532 and evaluating its potential anti-cancer activities (26). In SCCF1, SCCF2, and SCCF3, treatment with BIBR1532 caused the stoppage of cell growth in a dose-dependent manner, with a trend closely and specifically correlated to the degree of enzymatic inhibition, suggesting that FOSCC cells are telomerase-addicted in their proliferative abilities. Instead, despite with a general dose-dependent trend, cell viability was affected at different degrees unrelated to the levels of telomerase inhibition, particularly in SCCF3; a similar variable response has been reported in SCCFs treated with other chemotherapeutics, suggesting that this parameter may be specifically influenced by the molecular/metabolic background of each cell line (42): in agreement with this hypothesis, previous studies on breast cancer showed that different cell lines, even when derived from the same type of tumor, may exhibit distinct survival responses when treated with BIBR1532 (43). Additionally, morphological changes observed in treated SCCFs suggested the activation of replicative senescence: indeed, they are all distinctive features associated with age-related senescence and have been previously described in human cancer cells treated with BIBR1532 (29, 44).

Replicative senescence is triggered by telomeres attrition through up-regulation of the cell cycle inhibitor p21, and optionally, it may shift to apoptosis through unbalancing of Bax/Bcl-2 ratio (1). In SCCF1, molecular, biochemical, and cellular data indicated that, as the BIBR1532 dose increased, cells shifted from a p21-dependent cell cycle arrest to apoptosis. Studies on kinetics of apoptosis suggest that the apparent inconsistency of molecular results at 100 μM might be due to the fact that gene expression changes and effective cell death may be uncoupled, with the peak of Bax/Bcl-2 ratio possibly occurring prior to activation of Caspase 3 and apoptosis (45). Instead, in SCCF2, full consistency among all the experimental data revealed induction of cell cycle arrest and apoptosis with a dose-dependent trend. Regulation of p21, Bax, and Bcl-2 in response to telomerase inhibition requires an intact p53 pathway (1, 46); previously, SCCF3 cells were shown to harbor lower p53 levels than SCCF2, due to proteasomal degradation induced by *Felis catus* Papillomavirus type-2 E6, thus providing a likely explanation to the different degrees of activation of downstream genes between the two cell lines (47). However, in our recent work, SCCF3 cells were described to harbor lower basal TA, and this may justify the finding that the lowest BIBR1532 concentration was already sufficient to significantly inhibit the enzyme and result in earlier response of p21 (26). Nevertheless, the milder increase yielded by p21 in SCCF3 was coupled with a more impressive cell growth inhibition at each dose, suggesting that these cells were strictly p21-addicted for cell cycle arrest. BIBR1532 impacts on cell survival also through the down-regulation of Survivin in leukemia cells, although through unknown mechanisms (37, 38); our results suggest that Survivin cooperated in inducing apoptosis in SCCF1 and SCCF2 but not in SCCF3, where Caspase 3 cleavage and subsequent apoptosis at 100 μM might instead be due to the activation of alternative cell death mechanisms triggered by telomerase deprivation, such as the extrinsic pathway (48).

Taken together, these data suggest that inhibition of TA by BIBR1532 in SCCF cells switches off cell proliferation and, despite with variable degrees and molecular responses, promotes cell death mostly by engaging p53-dependent pathways triggered by telomeres shortening, in agreement with literature data (12, 49–51). However, several authors found that acute treatment with BIBR1532 is not sufficient to obtain a substantial telomeres attrition; instead, it induces direct random DNA damage, likely due to the inhibition of extra-telomeric functions of TERT in DNA repair processes rather than to disable its canonical activity at telomeres (12). Moreover, in FOSCC samples, lower TA does not correspond to shorter telomeres, possibly due to the activation of mechanisms of alternative lengthening of telomeres (ALT) (20). In this study, we did not measure telomeres length upon treatment and cannot exclude the occurrence of ALT in SCCF cells; however, our data demonstrate that, whatever the cellular and molecular mechanisms involved, BIBR1532 exerts potent tumor growth suppressive activities in FOSCC (see Supplementary Figure 5).

TERT gene is under transcriptional regulation by cMyc, a well-known oncogene over-expressed in FOSCC and previously detected in SCCF cells (3, 26, 52). BIBR1532 down-regulates cMyc expression in tumor cell lines through the perturbation of regulatory cancer-related microRNAs (miRNA), resulting in the repression of TERT expression and subsequent inhibition of its extra-telomeric functions (36–39). In this work, SCCF cells treated with BIBR1532 showed concomitant down-regulation of cMyc and TERT, suggesting the occurrence of this transcriptional axis in FOSCC. Whether this is due to the dysregulation of miRNA induced by BIBR1532 also in FOSCC, needs to be explored in future studies.

Regulation of EGFR is among the non-canonical functions of TERT (2), and interestingly, previous studies report a significant correlation between EGFR expression and levels of TA in FOSCC samples (20). Consistently, here, BIBR1532 induced down-regulation of EGFR and its downstream effector pERK in all cell lines, confirming its functional correlation with TERT in FOSCC also *in vitro*. Considering the main role of EGFR in the proliferation of FOSCC-derived cells (40), it is likely that the inhibition of EGFR in treated SCCF cells contributed to the reduction of their growth; therefore, our data pave the way to a possible dual-target therapeutic approach blocking two key pathways in the development of FOSCC by the use of a single drug, the BIBR1532.

FOSCC is characterized by highly invasive phenotype and metastatic potential, possibly due to the expression of MMP-1/-2 and−9 as in the human counterpart (26). It has been demonstrated that modulation of MMPs expression is an additional extra-telomeric function of TERT, so that its specific inhibition causes a down-regulation of MMPs accompanied by a decrease in invasion capacity of cell lines derived from different tumors, including hOSCC (8, 9, 17). In agreement with these data, inhibition of TERT by BIBR1532 in SCCFs induced a reduction of MMP-1/-2/-9 expression, suggesting that treatment with this drug may affect also the invasive potential of tumor cells in FOSCC.

In conclusion, this work demonstrates that BIBR1532 exerts a series of anti-cancer activities linked to the inhibition of canonical telomerase pathway and TERT extra-telomeric functions in FOSCC cell lines (see Supplementary Figure 5), paving the way for future translational studies aimed at evaluating its possible employment in the treatment of this tumor. There is evidence showing that BIBR1532 works synergistically with chemotherapeutic agents and ionizing radiation, sensitizing drug-resistant cells and enhancing radio-sensitivity in many *in vitro* models of cancer (14, 38, 49, 53). Considering that chemo- and radiotherapy are not resolutive in FOSCC patients, we may speculate that BIBR1532 may find potential application as an adjunctive agent contributing to improve the response to treatments, leading to a more favorable prognosis for naturally occurring FOSCC.

## Data Availability Statement

The original contributions presented in the study are included in the article/Supplementary Material, further inquiries can be directed to the corresponding author/s.

## Author Contributions

GA, LL, KP, and GDL performed the experiments. GA, GB, and PM drafted the manuscript. GA, GDL, PM, GG, BDU, and GB conceived the study. All authors approved the submitted version.

## Conflict of Interest

The authors declare that the research was conducted in the absence of any commercial or financial relationships that could be construed as a potential conflict of interest.

## References

[1] Deng Y Chan SS Chang S. Telomere dysfunction and tumour suppression: the senescence connection. Nat Rev Cancer. (2008) 8:450–8. 10.1038/nrc2393 18500246 PMC3688269

[2] Low KC Tergaonkar V. Telomerase: central regulator of all of the hallmarks of cancer. Trends Biochem Sci. (2013) 38:426–34. 10.1016/j.tibs.2013.07.001 23932019

[3] Cerni C. Telomeres, telomerase, and myc. An update. Mutat Res. (2000) 462:31–47. 10.1016/s1383-5742(99)00091-5 10648922

[4] McMurray HR McCance DJ. Human papillomavirus type 16 E6 activates TERT gene transcription through induction of c-Myc and release of USF-mediated repression. J Virol. (2003) 77:9852–61. 10.1128/jvi.77.18.9852-9861.2003 12941894 PMC224601

[5] Smith LL Coller HA Roberts JM. Telomerase modulates expression of growth-controlling genes and enhances cell proliferation. Nat Cell Biol. (2003) 5:474–9. 10.1038/ncb985 12717449

[6] Beck S Jin X Sohn YW Kim JK Kim SH Yin J. Telomerase activity-independent function of TERT allows glioma cells to attain cancer stem cell characteristics by inducing EGFR expression. Mol Cells. (2011) 31:9–15. 10.1007/s10059-011-0008-8 21193962 PMC3906874

[7] Ding D Xi P Zhou J Wang M Cong YS. Human telomerase reverse transcriptase regulates MMP expression independently of telomerase activity via NF-kappaB-dependent transcription. FASEB J. (2013) 27:4375–83. 10.1096/fj.13-230904 23884427

[8] Kong W Lv N Wysham WZ Roque DR Zhang T Jiao S. Knockdown of hTERT and treatment with BIBR1532 inhibit cell proliferation and invasion in endometrial cancer cells. J Cancer. (2015) 6:1337–45. 10.7150/jca.13054 26640594 PMC4643090

[9] Kusoglu A Goker Bagca B Ozates Ay NP Gunduz C Biray Avci. C. Telomerase inhibition regulates EMT mechanism in breast cancer stem cells. Gene. (2020) 759:145001. 10.1016/j.gene.2020.145001 32738420

[10] Saraswati AP Relitti N Brindisi M Gemma S Zisterer D Butini S. Raising the bar in anticancer therapy: recent advances in, perspectives on telomerase inhibitors. Drug Discov Today. (2019) 24:1370–88. 10.1016/j.drudis.2019.05.015 31136800

[11] Bryan C Rice C Hoffman H Harkisheimer M Sweeney M Skordalakes E. Structural basis of telomerase inhibition by the highly specific BIBR1532. Structure. (2015) 23:1934–42. 10.1016/j.str.2015.08.006 26365799 PMC4598299

[12] Celeghin A Giunco S Freguja R Zangrossi M Nalio S Dolcetti R. Short-term inhibition of TERT induces telomere length-independent cell cycle arrest and apoptotic response in EBV-immortalized and transformed B cells. Cell Death Dis. (2016) 7:e2562. 10.1038/cddis.2016.425 28032863 PMC5260987

[13] Bashash D Ghaffari SH Zaker F Kazerani M Hezave K Hassani S. BIBR 1532 increases arsenic trioxide-mediated apoptosis in acute promyelocytic leukemia cells: therapeutic potential for APL. Anticancer Agents Med Chem. (2013) 13:1115–1125. 10.2174/18715206113139990126 23293885

[14] Ding X Cheng J Pang Q Wei X Zhang X Wang P. BIBR1532, a selective telomerase inhibitor, enhances radiosensitivity of non-small cell lung cancer through increasing telomere dysfunction and ATM/CHK1 inhibition. Int J Radiat Oncol Biol Phys. (2019) 105:861–74. 10.1016/j.ijrobp.2019.08.009 31419512

[15] Nasrollahzadeh A Bashash D Kabuli M Zandi Z Kashani B Zaghal A. Arsenic trioxide and BIBR1532 synergistically inhibit breast cancer cell proliferation through attenuation of NF-kappaB signaling pathway. Life Sci. (2020) 257:118060. 10.1016/j.lfs.2020.118060 32645343

[16] Chen HH Yu CH Wang JT Liu BY Wang YP Sun A. Expression of human telomerase reverse transcriptase (hTERT) protein is significantly associated with the progression, recurrence and prognosis of oral squamous cell carcinoma in Taiwan. Oral Oncol. (2007) 43:122–29. 10.1016/j.oraloncology.2006.01.011 16798059

[17] Park YJ Kim EK Moon S Hong DP Bae JY Kim J. Human telomerase reverse transcriptase is a promising target for cancer inhibition in squamous cell carcinomas. Anticancer Res. (2014) 34:6389–95. 25368238

[18] Raghunandan BN Sanjai K Kumaraswamy J Papaiah L Pandey B Jyothi BM. Expression of human telomerase reverse transcriptase protein in oral epithelial dysplasia and oral squamous cell carcinoma: an immunohistochemical study. J Oral Maxillofac Pathol. (2016) 20:96–101. 10.4103/0973-029X.180953 27194869 PMC4860945

[19] Pang LY Argyle DJ. Using naturally occurring tumours in dogs and cats to study telomerase and cancer stem cell biology. Biochim Biophys Acta. (2009) 1792:380–91. 10.1016/j.bbadis.2009.02.010 19254761

[20] Yoshikawa H Maranon DG Battaglia CLR Ehrhart EJ Charles JB Bailey SM. Predicting clinical outcome in feline oral squamous cell carcinoma: tumour initiating cells, telomeres and telomerase. Vet Comp Oncol. (2016) 14:371–83. 10.1111/vco.12117 25212092

[21] Thalmensi J Pliquet E Liard C Chamel G Kreuz C Bestetti T. A DNA telomerase vaccine for canine cancer immunotherapy. Oncotarget. (2019) 10:3361–72. 10.18632/oncotarget.26927 31164958 PMC6534364

[22] Supsavhad W Dirksen WP Martin CK Rosol TJ. Animal models of head and neck squamous cell carcinoma. Vet J. (2016) 210:7–16. 10.1016/j.tvjl.2015.11.006 26965084

[23] Bilgic O Duda L Sanchez MD Lewis JR. Feline oral squamous cell carcinoma: clinical manifestations and literature review. J Vet Dent. (2015) 32:30–40. 10.1177/089875641503200104 26197688

[24] Martin CK Werbeck JL Thudi NK Lanigan LG Wolfe TD Toribio RE. Zoledronic acid reduces bone loss and tumor growth in an orthotopic xenograft model of osteolytic oral squamous cell carcinoma. Cancer Res. (2010) 70:8607–16. 10.1158/0008-5472.CAN-10-0850 20959474 PMC2970642

[25] Martin CK Dirksen WP Shu ST Werbeck JL Thudi NK Yamaguchi M. Characterization of bone resorption in novel *in vitro* and *in vivo* models of oral squamous cell carcinoma. Oral Oncol. (2012) 48:491–9. 10.1016/j.oraloncology.2011.12.012 22265717 PMC3350825

[26] Altamura G Martano M Licenziato L Maiolino P Borzacchiello G. Telomerase reverse transcriptase (TERT) expression, telomerase activity, and expression of matrix metalloproteinases (MMP)-1/-2/-9 in feline oral squamous cell carcinoma cell lines associated with felis catus papillomavirus type-2 infection. Front Vet Sci. (2020) 7:148. 10.3389/fvets.2020.00148 32292795 PMC7118734

[27] Tannehill-Gregg S Kergosien E Rosol TJ. Feline head and neck squamous cell carcinoma cell line: characterization, production of parathyroid hormone-related protein, and regulation by transforming growth factor-beta. In Vitro Cell Dev Biol Anim. (2001) 37:676–683. 10.1290/1071-2690(2001)037<0676:FHANSC>2.0.CO;2 11776973

[28] Altamura G Corteggio A Nasir L Yuan ZQ Roperto F Borzacchiello G. Analysis of activated platelet-derived growth factor beta receptor and Ras-MAP kinase pathway in equine sarcoid fibroblasts. Biomed Res Int. (2013) 2013:283985. 10.1155/2013/283985 23936786 PMC3726019

[29] Altamura G Corteggio A Pacini L Conte A Pierantoni GM Tommasino M. Transforming properties of Felis catus papillomavirus type 2 E6 and E7 putative oncogenes *in vitro* and their transcriptional activity in feline squamous cell carcinoma *in vivo*. Virology. (2016) 496:1–8. 10.1016/j.virol.2016.05.017 27236740

[30] Altamura G Uberti BD Galiero G Martano M Pirro A Russo M. Expression and activation of platelet-derived growth factor beta receptor, mitogen-activated protein/extracellular signal-regulated kinase kinase (MEK) and extracellular signal-regulated kinase (ERK) in canine mammary tumours. Res Vet Sci. (2017) 110:29–33. 10.1016/j.rvsc.2016.10.014 28159233

[31] Altamura G Corteggio A Borzacchiello G. Felis catus papillomavirus type 2 E6 oncogene enhances mitogen-activated protein kinases and Akt activation but not EGFR expression in an *in vitro* feline model of viral pathogenesis. Vet Microbiol. (2016) 195:96–100. 10.1016/j.vetmic.2016.09.013 27771077

[32] Sano J Nagafuchi S Yamazaki J Oguma K Kano R Hasegawa A. Effect of antineoplastic drugs on the expression of Bcl-2 and Bcl-xL genes in the feline T-cell leukemia cell line. Res Vet Sci. (2005) 79:197–201. 10.1016/j.rvsc.2005.03.001 15893350

[33] Mochizuki H Goto-Koshino Y Sato M Fujino Y Ohno K Tsujimoto H Comparison of the antitumor effects of an MDM2 inhibitor, nutlin-3, in feline lymphoma cell lines with or without p53 mutation. Vet Immunol Immunopathol. (2012) 147:187–94. 10.1016/j.vetimm.2012.04.017 22578852

[34] Brown ME Bear MD Rosol TJ Premanandan C Kisseberth WC London CA. Characterization of STAT3 expression, signaling and inhibition in feline oral squamous cell carcinoma. BMC Vet Res. (2015) 11:206. 10.1186/s12917-015-0505-7 26272737 PMC4536595

[35] Penning LC Vrieling HE Brinkhof B Riemers FM Rothuizen J Rutteman GR. A validation of 10 feline reference genes for gene expression measurements in snap-frozen tissues. Vet Immunol Immunopathol. (2007) 120:212–22. 10.1016/j.vetimm.2007.08.006 17904230

[36] Bashash D Ghaffari SH Zaker F Hezave K Kazerani M Ghavamzadeh A. Direct short-term cytotoxic effects of BIBR 1532 on acute promyelocytic leukemia cells through induction of p21 coupled with downregulation of c-Myc and hTERT transcription. Cancer Invest. (2012) 30:57–64. 10.3109/07357907.2011.629378 22236190

[37] Bashash D Ghaffari SH Mirzaee R Alimoghaddam K Ghavamzadeh A. Telomerase inhibition by non-nucleosidic compound BIBR1532 causes rapid cell death in pre-B acute lymphoblastic leukemia cells. Leuk Lymphoma. (2013) 54:561–8. 10.3109/10428194.2012.704034 22957790

[38] Bashash D Zareii M Safaroghli-Azar A Omrani MD Ghaffari SH. Inhibition of telomerase using BIBR1532 enhances doxorubicin-induced apoptosis in pre-B acute lymphoblastic leukemia cells. Hematology. (2017) 22:330–40. 10.1080/10245332.2016.1275426 28054503

[39] Pourbagheri-Sigaroodi A Bashash D Safaroghli-Azar A Farshi-Paraasghari M Momeny M Mansoor FN. Contributory role of microRNAs in anti-cancer effects of small molecule inhibitor of telomerase (BIBR1532) on acute promyelocytic leukemia cell line. Eur J Pharmacol. (2019) 846:49–62. 10.1016/j.ejphar.2019.01.018 30658112

[40] Bergkvist GT Argyle DJ Pang LY Muirhead R Yool DA. Studies on the inhibition of feline EGFR in squamous cell carcinoma: enhancement of radiosensitivity and rescue of resistance to small molecule inhibitors. Cancer Biol Ther. (2011) 11:927–37. 10.4161/cbt.11.11.15525 21464610

[41] Schlessinger J. Cell signaling by receptor tyrosine kinases. Cell. (2000) 103:211–25. 10.1016/s0092-8674(00)00114-8 11057895

[42] Piegols HJ Takada M Parys M Dexheimer T Yuzbasiyan-Gurkan V. Investigation of novel chemotherapeutics for feline oral squamous cell carcinoma. Oncotarget. (2018) 9:33098–109. 10.18632/oncotarget.26006 30237854 PMC6145701

[43] Dogan F Ozates NP Bagca BG Abbaszadeh Z Sogutlu F Gasimli R. Investigation of the effect of telomerase inhibitor BIBR1532 on breast cancer and breast cancer stem cells. J Cell Biochem. (2018) 120:1282–93. 10.1002/jcb.27089 30368861

[44] Damm K Hemmann U Garin-Chesa P Hauel N Kauffmann I Priepke H. A highly selective telomerase inhibitor limiting human cancer cell proliferation. EMBO J. (2001) 20:6958–68. 10.1093/emboj/20.24.6958 11742973 PMC125790

[45] Chen J Li X Qiu J You H Zhang Q Dong G. Kinetics of apoptosis and expression of apoptosis-related proteins in rat CA3 hippocampus cells after experimental diffuse brain injury. Cell Biochem Biophys. (2013) 67:1015–9. 10.1007/s12013-013-9597-5 23559276 PMC3838593

[46] Brassat U Balabanov S Bali D Dierlamm J Braig M Hartmann U. Functional p53 is required for effective execution of telomerase inhibition in BCR-ABL-positive CML cells. Exp Hematol. (2011) 39:66–76 e61–62. 10.1016/j.exphem.2010.10.001 20940029

[47] Altamura G Power K Martano M Degli Uberti B Galiero G De Luca G. Felis catus papillomavirus type-2 E6 binds to E6AP, promotes E6AP/p53 binding and enhances p53 proteasomal degradation. Sci Rep. (2018) 8:17529. 10.1038/s41598-018-35723-7 30510267 PMC6277439

[48] Ren X Zhang Z Tian J Wang H Song G Guo Q. The downregulation of c-Myc and its target gene hTERT is associated with the antiproliferative effects of baicalin on HL-60 cells. Oncol Lett. (2017) 14:6833–6840. 10.3892/ol.2017.7039 29163703 PMC5688794

[49] Ward RJ Autexier C. Pharmacological telomerase inhibition can sensitize drug-resistant and drug-sensitive cells to chemotherapeutic treatment. Mol Pharmacol. (2005) 68:779–86. 10.1124/mol.105.011494 15939802

[50] Parsch D Brassat U Brummendorf TH Fellenberg J. Consequences of telomerase inhibition by BIBR1532 on proliferation and chemosensitivity of chondrosarcoma cell lines. Cancer Invest. (2008) 26:590–6. 10.1080/07357900802072905 18584350

[51] Nakashima M Nandakumar J Sullivan KD Espinosa JM Cech TR. Inhibition of telomerase recruitment and cancer cell death. J Biol Chem. (2013) 288:33171–80. 10.1074/jbc.M113.518175 24097987 PMC3829164

[52] Giuliano A Swift R Arthurs C Marote G Abramo F McKay J. Quantitative expression and co-localization of Wnt signalling related proteins in feline squamous cell carcinoma. PLoS ONE. (2016) 11:e0161103. 10.1371/journal.pone.0161103 27559731 PMC4999089

[53] Shi Y Sun L Chen G Zheng D Li L Wei W. A combination of the telomerase inhibitor, BIBR1532, and paclitaxel synergistically inhibit cell proliferation in breast cancer cell lines. Target Oncol. (2015) 10:565–73. 10.1007/s11523-015-0364-y 25916999

