# Supplementary material for: The Small Molecule BIBR1532 Exerts Potential Anti-cancer Activities in Preclinical Models of Feline Oral Squamous Cell Carcinoma Through Inhibition of Telomerase Activity and Down-Regulation of TERT
Source: Front Vet Sci. 2021 Jan 20;7:620776. doi: 10.3389/fvets.2020.620776 (PMC7855307; doi:10.3389/fvets.2020.620776)
Supplement: Supplementary file 1 [file Data_Sheet_1.PDF]

Supplementary Material

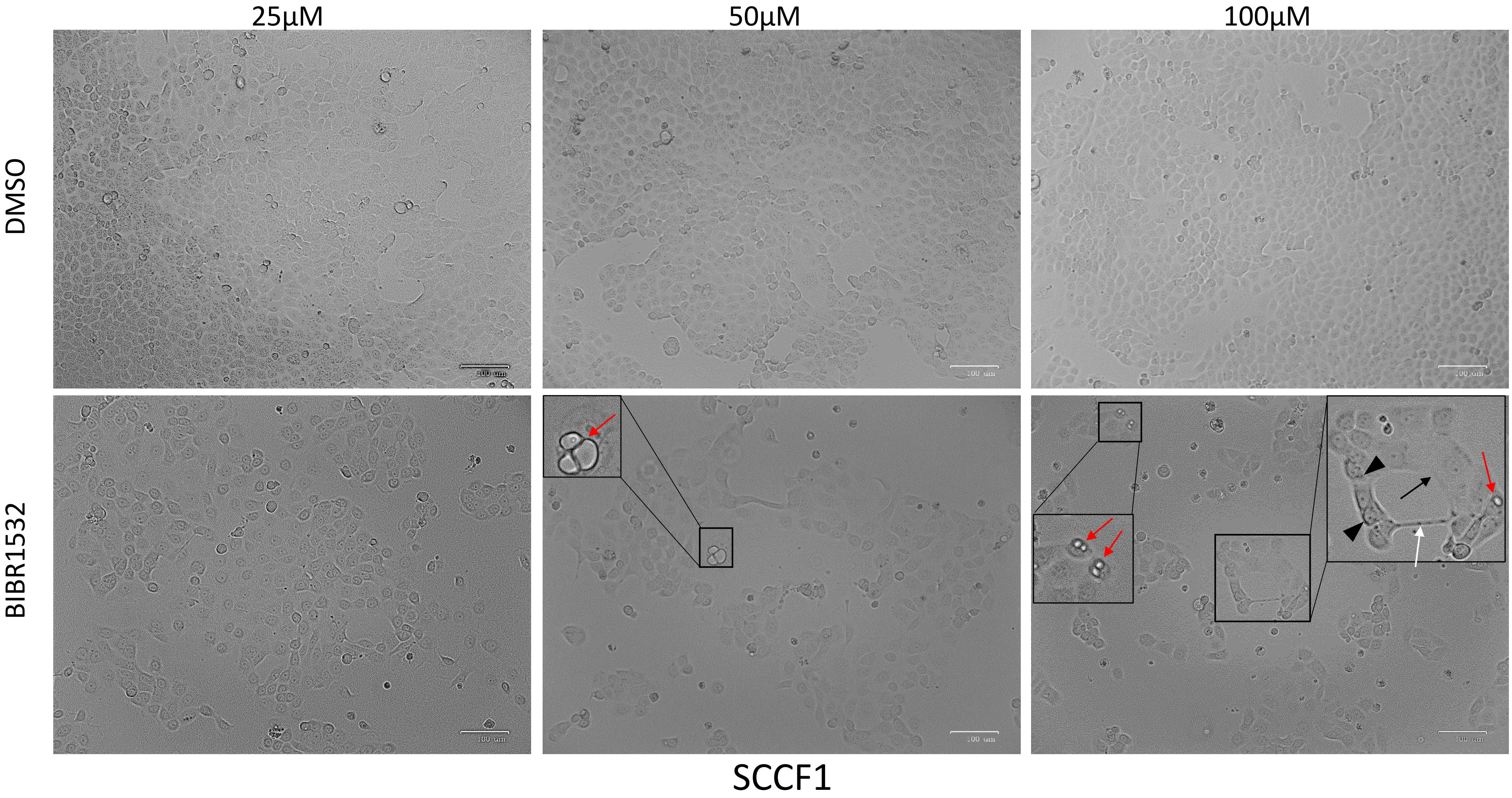

Supplementary Figure S1A

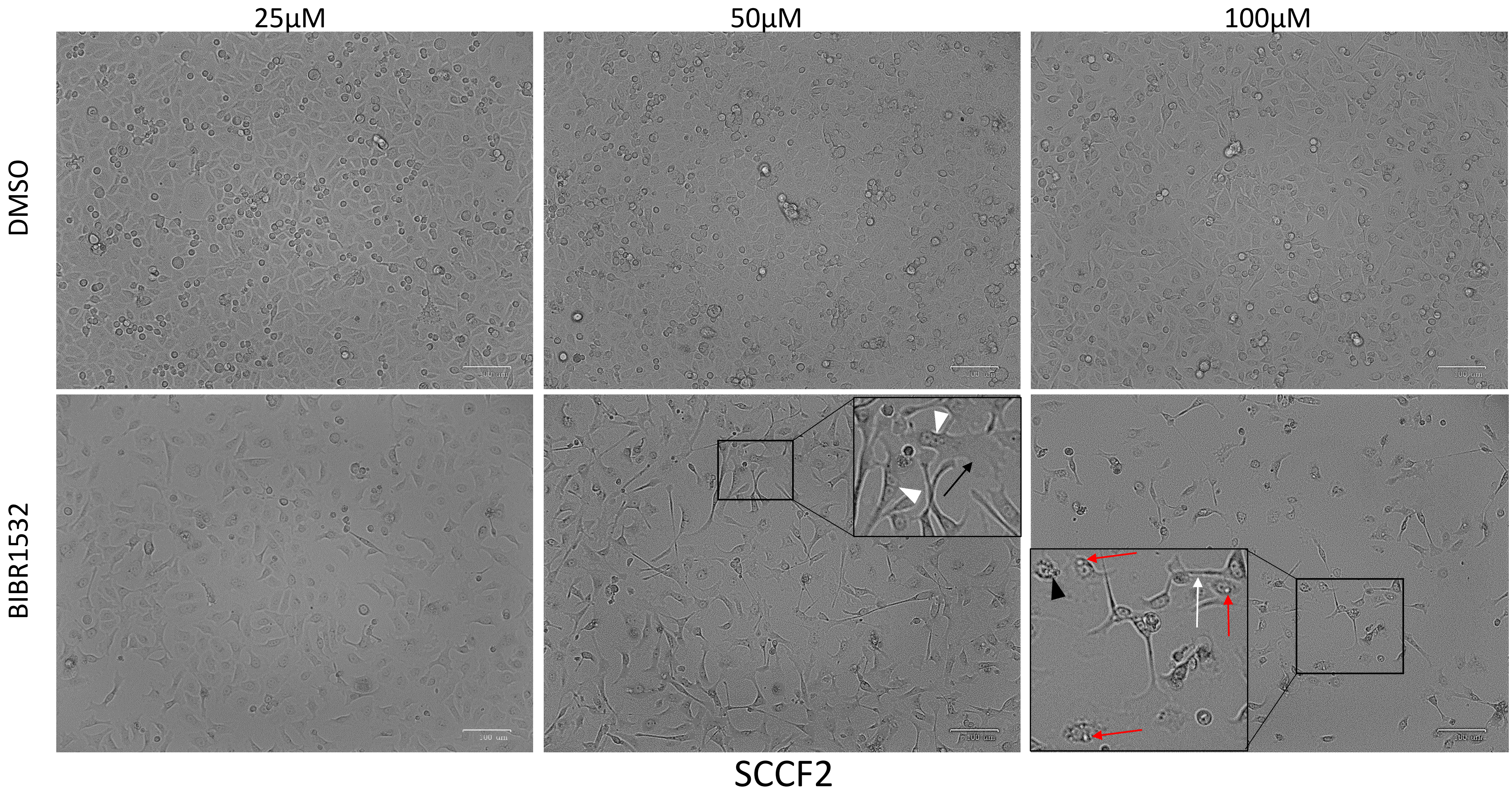

Supplementary Figure S1B

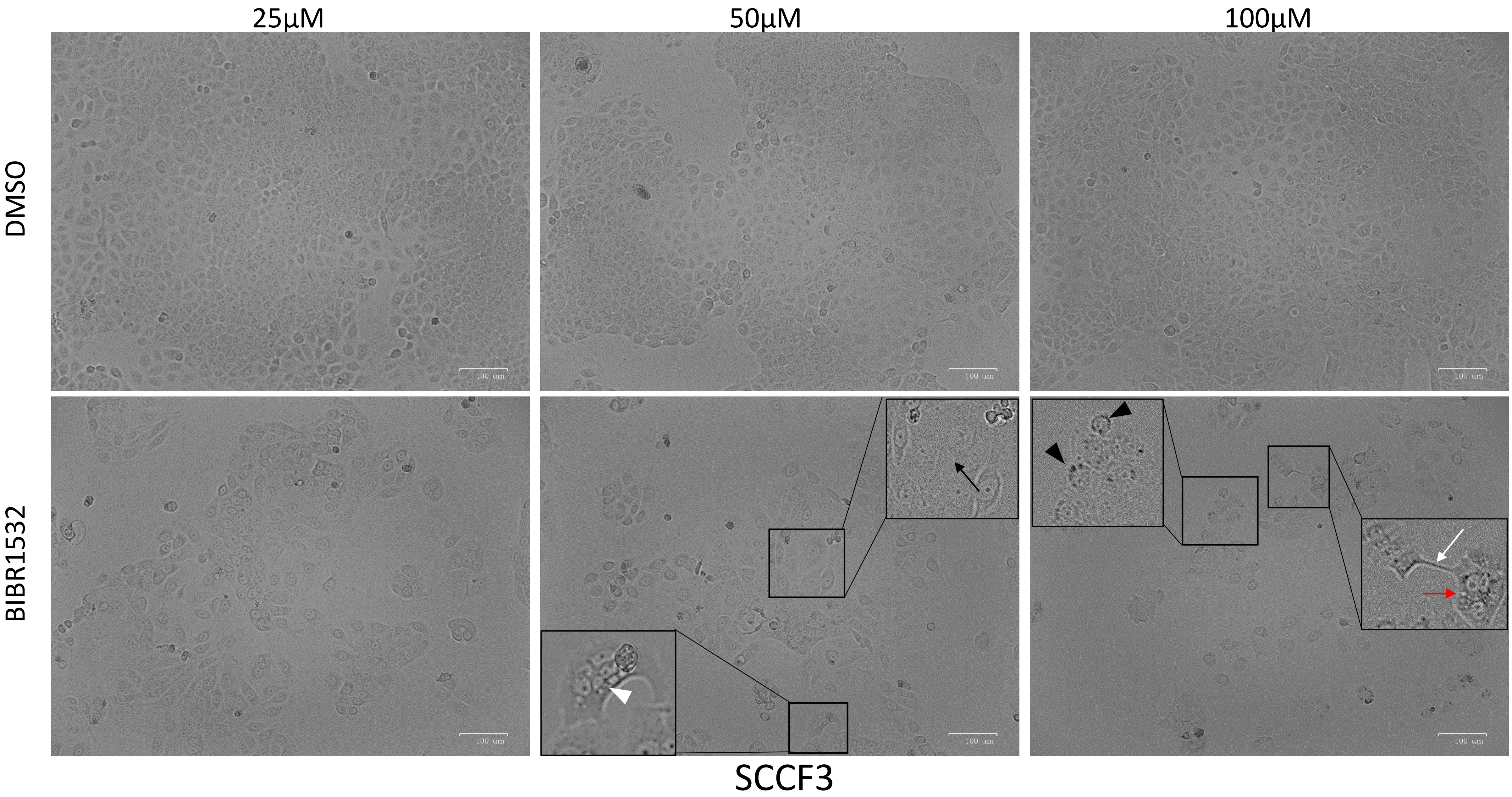

Supplementary Figure S1C

Supplementary Figure S1. BIBR1532 induces morphological changes resembling a senescent phenotype. SCCF1 **(A)**, SCCF2 **(B)** and SCCF3 **(C)** were treated with BIBR1532 at 25, 50 and 100µM *vs* DMSO and scanned by phase contrast microscope. Unlike untreated cells, growth-arrested SCCFs became flattened and enlarged (black arrows), with intercellular bridges (white arrows) and increased granularity (black arrowheads), often multi-nucleated (white arrowheads) and had vacuolated cytoplasm (red arrowheads). Representative fields are shown.

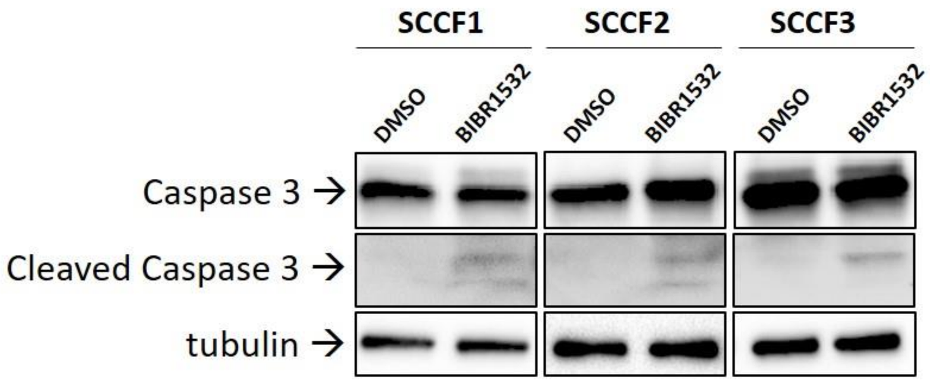

Supplementary Figure S2. BIBR1532 induces apoptosis in SCCF1, SCCF2 and SCCF3. Western blotting showing cleavage of Caspase 3 as marker of apoptosis in cells treated with BIBR1532 at 100µM for 48h. Blot was stripped and reprobed for tubulin as loading control.

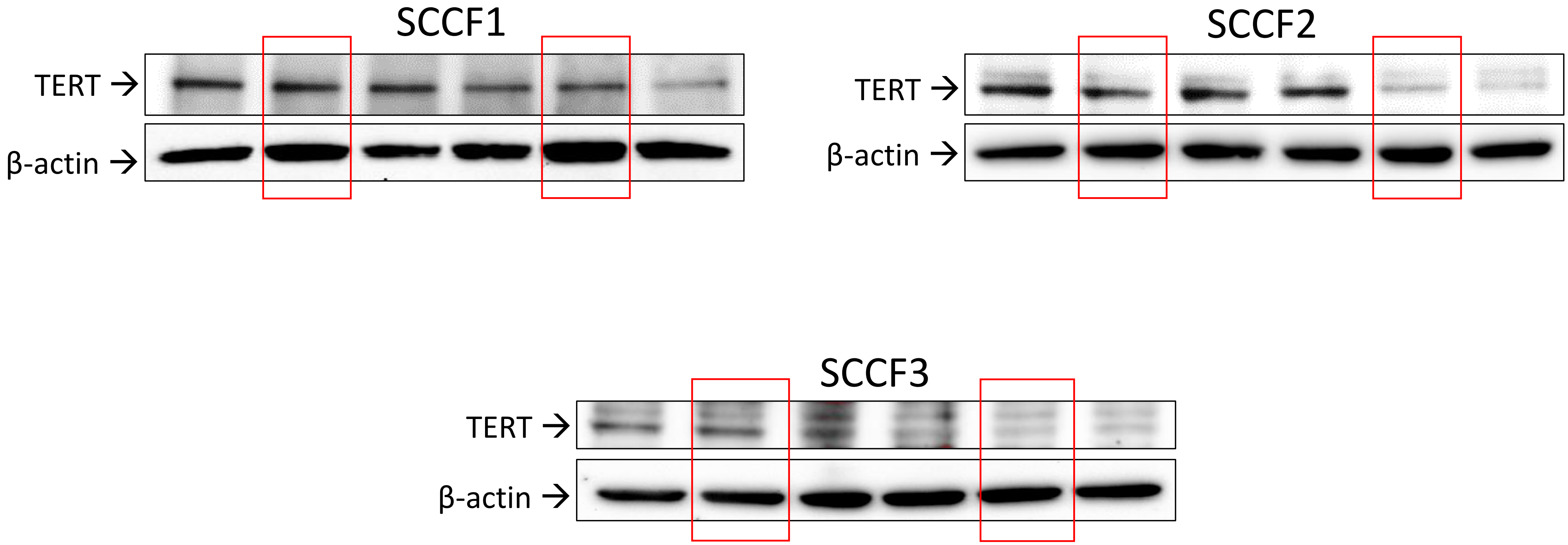

Supplementary Figure S3. Full length blots from original gels of Western blotting experiments for TERT shown in Figure 4. Red rectangles indicate the samples included in the figure.

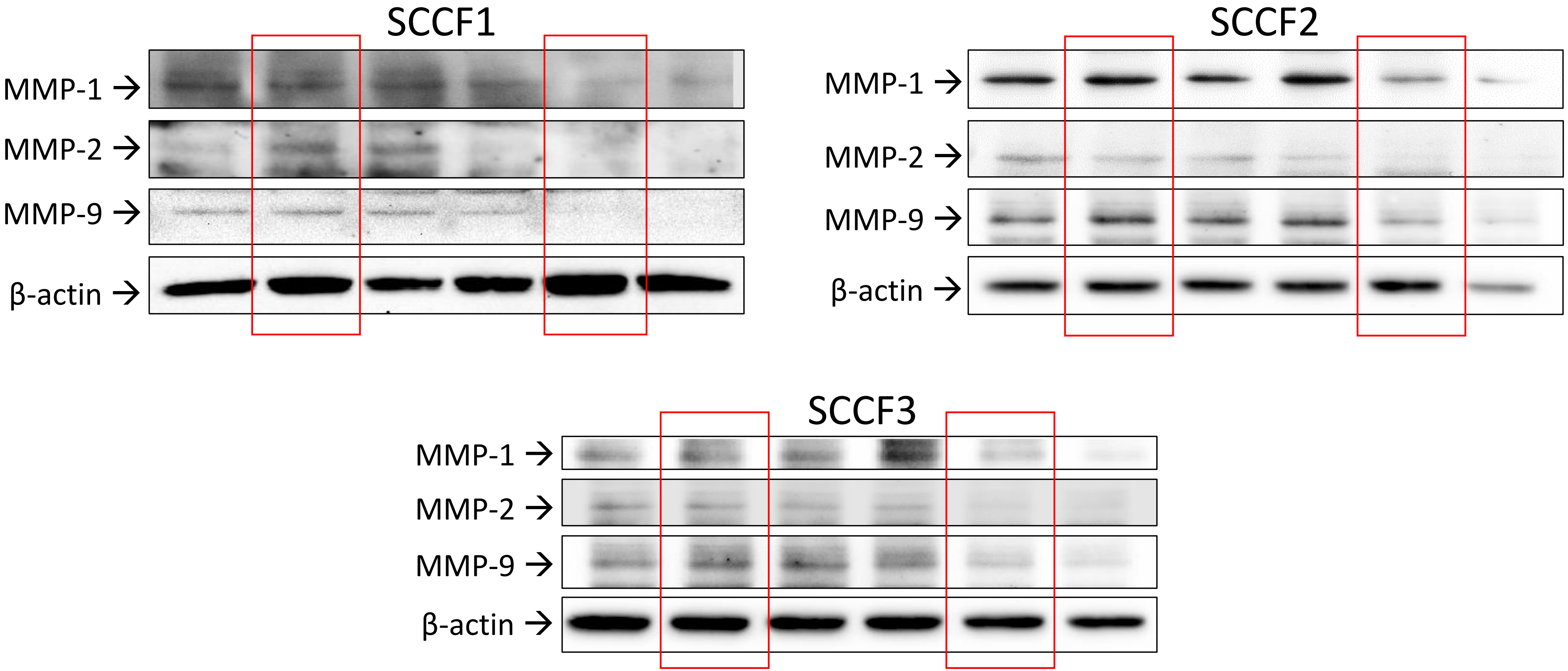

Supplementary Figure S4. Full length blots from original gels of Western blotting experiments for MMP-1/-2/-9 shown in Figure 6. Red rectangles indicate the samples included in the figure.

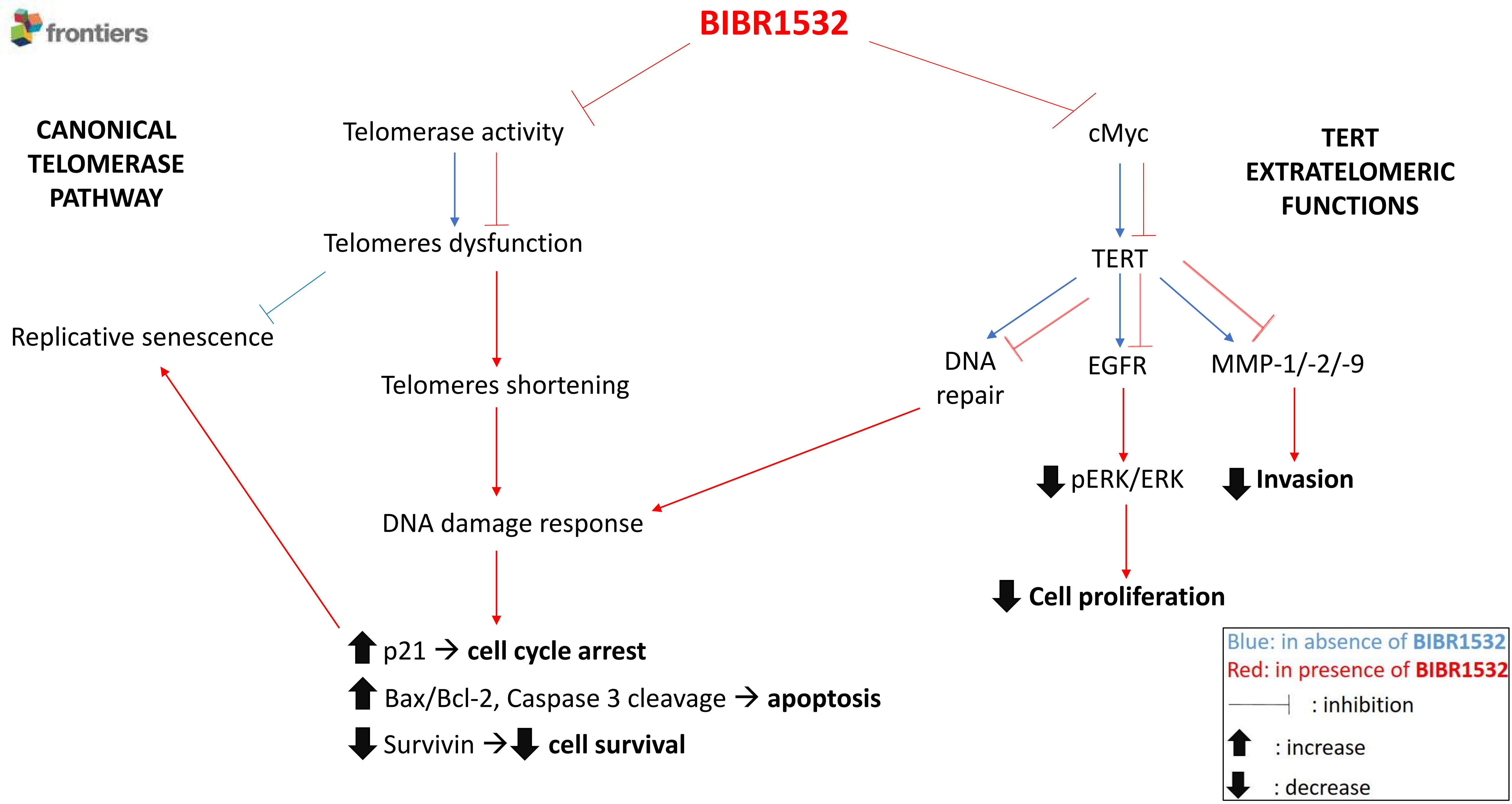

Supplementary Figure S5. Schematic representation of the results obtained in this work and the possible underlying mechanisms.
